# A functionalized enol lactone containing a protected α-amino acid

**DOI:** 10.1107/S1600536810043709

**Published:** 2010-11-30

**Authors:** Giuseppe Bruno, Francesco Nicoló, Massimiliano Cordaro, Giovanni Grassi, Francesco Risitano

**Affiliations:** a Dipartimento di Chimica Inorganica Chimica Analitica e Chimica Fisica, Universitá degli Studi di Messina, Via Salita Sperone 31, I-98166 Vill. S. Agata, Messina, Italy; b Dipartimento di Chimica Organica e Biologica, Universitá degli Studi di Messina, Via Salita Sperone 31, I-98166 Vill. S. Agata, Messina, Italy

## Abstract

The crystal structure of *N*-(3,9-dimethyl-4-phenyl-2,5-dioxo-3,4-dihydro-2*H*,5*H*-pyrano[3,2-*c*]chromen-3-yl)-*N*-methylbenzamide methanol monosolvate, C_28_H_23_NO_5_·CH_3_OH, has been determined at room temperature by X-ray diffraction. Structural parameters are discussed with reference to *ab initio* calculations.

## Related literature

For structures containing enol lactone fragments, see: Murray *et al.* (1982 ▶); Harborne & Baxter (1999 ▶); Qabaja *et al.* (2000 ▶). For related structures containing a coumarin fragment, see: Yu *et al.* (2003 ▶). For a phenyl­furo[3,2-*c*]chromen-4-one structure, see: Bruno *et al.* (2001 ▶). For related furan-5-one structures, see: Grassi *et al.* (2002 ▶). For the Cambridge Structural Database, see: Allen (2002 ▶). For the *GAUSSIAN98* software used for the ab initio calculations, see: Frisch *et al.* (1998 ▶). For the synthetic process used, see: Grassi *et al.* (2003 ▶). For background to O—C—O bond-angle asymmetry, see: Kokila *et al.* (1996 ▶).
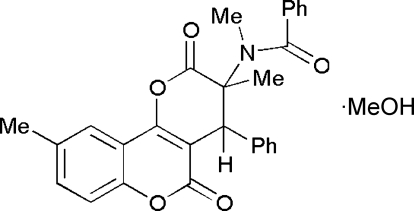


## Experimental

### 

#### Crystal data

C_28_H_23_NO_5_·CH_4_O
*M* *_r_* = 485.52
Monoclinic, 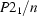

*a* = 10.7435 (14) Å
*b* = 9.9056 (17) Å
*c* = 23.298 (3) Å
β = 94.817 (7)°
*V* = 2470.7 (6) Å^3^
*Z* = 4
Mo *K*α radiation
μ = 0.09 mm^−1^
*T* = 298 K
0.58 × 0.38 × 0.36 mm

#### Data collection

Siemens *P*4 diffractometer
Absorption correction: ψ scan (Kopfmann & Huber, 1968 ▶) *T*_min_ = 0.870, *T*_max_ = 0.968
9535 measured reflections
4347 independent reflections
2843 reflections with *I* > 2σ(*I*)
*R*_int_ = 0.036
3 standard reflections every 197 reflections  intensity decay: none

#### Refinement

*R*[*F* ^2^ > 2σ(*F* ^2^)] = 0.049
*wR*(*F* ^2^) = 0.132
*S* = 1.01
4347 reflections
331 parameters
H-atom parameters constrained
Δρ_max_ = 0.18 e Å^−3^
Δρ_min_ = −0.31 e Å^−3^

### 

Data collection: *XSCANS* (Bruker, 1999 ▶); cell refinement: *XSCANS*; data reduction: *SHELXTL* (Sheldrick, 2008 ▶); program(s) used to solve structure: *SIR97* (Altomare *et al.*, 1999 ▶); program(s) used to refine structure: *SHELXL97* (Sheldrick, 2008 ▶); molecular graphics: XPW (Siemens, 1996 ▶); software used to prepare material for publication: *PARST97* (Nardelli, 1995 ▶) and *SHELXL97*.

## Supplementary Material

Crystal structure: contains datablocks global, I. DOI: 10.1107/S1600536810043709/jh2217sup1.cif

Structure factors: contains datablocks I. DOI: 10.1107/S1600536810043709/jh2217Isup2.hkl

Additional supplementary materials:  crystallographic information; 3D view; checkCIF report

## Figures and Tables

**Table 1:** Hydrogen-bond geometry (Å, °)

*D*—H⋯*A*	*D*—H	H⋯*A*	*D*⋯*A*	*D*—H⋯*A*
O35—H35⋯O27	0.82	1.99	2.784 (3)	163
C9—H9⋯O35^i^	0.93	2.46	3.320 (4)	153
C19—H19⋯O11^ii^	0.93	2.60	3.403 (3)	144

Symmetry codes: (i) 
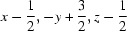
; (ii) 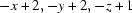.

**Fig. 1.**
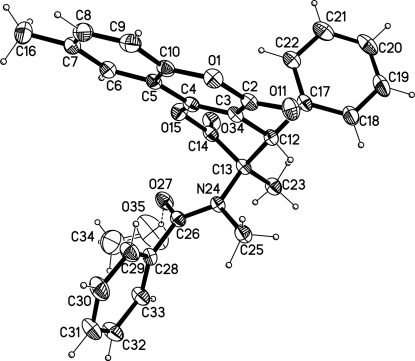
Perspective view of the asymmetric unit with numbering scheme of compound 3. Dashed line represents H-bond with the co-crystallized methanol hydrogen. Ellipsoids are drawn at 20% of probability level while hydrogen size is arbitrary.

**Fig. 2.**
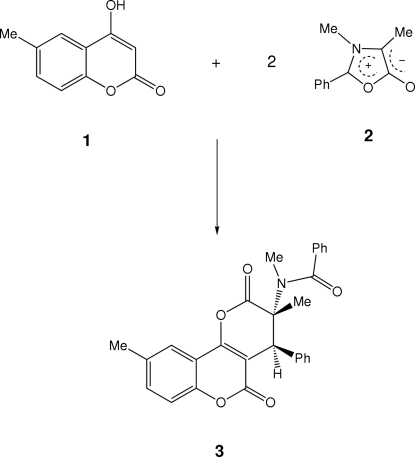
Reaction scheme.

## supplementary crystallographic information

### Comment

Highly functionalized enol lactones constitute an attractive class of compounds not only because of their molecular architecture but also because of the interesting biopharmacological activity often shown by compounds possessing this subunit in their molecular skeleton (Murray *et al.*, 1982; Harborne & Baxter, 1999; Qabaja *et al.*, 2000).

The molecule 3 is mainly constituted by a cumarine system which is further [3,2-*c*]-fused with a 3,4-dihydro-pyran-2-one ring carrying at posistion 3 a methyl and a methyllbenzamide group while a phenyl moiety is placed at 4. Then these C atoms at 3 and 4 are chiral centers showing the same configuration. Due to the *P*2_1_/*n* centric space group, both the *S*,*R* and *R*,*S* diastereomers are present in the solid state and the crystals represent a perfect racemic mixture. The methyl cumarin fragment is planar within experimental error; bonds distances and angles of this moiety are in good agreement with the corresponding values reported for such fragments (Yu *et al.*, 2003). The methylbenzamide fragment is not planar and shows the usual bond geometry values: the phenyl ring is rotated by 60.9 (3)° with respect to the amide moiety. The three carbonyl groups have very different C═O bond distances and their O atoms are involved in several intra- and intermolecular hydrogen interactions. The significant elongation of C(26)—O(27) bond might be related to the strongest hydrogen interaction of its O atom with the co-crystallized methanol molecule: O(27)···H(35) and O(27)···O(35) are 1.99 and 2.784 (3) Å, respectively, while O(35)—H(35)···O(27) is 163°. The sum of the valence angles around N(24) is 357.7 (2)° and evidences the not significant pyramidalization of the nitrogen. Therefore the nitrogen geometry appears planar and might be related to its *sp*^2^ hybridization caused by the delocalization of its lone-pair over the carbonyl fragments as also suggested by the length of the bonds involving the N(24) atom. Phenyl group bonded to C(26) is rotated with respect to this system (the N(24)—C(26)—C(28)—C(29) torsion angle is 60.5 (3)°) and does not participate to the possible π-electronic delocalization as evidenced by the C(28)—C(26) single-bond length value [1.491 (3) Å]. In the crystal lattice, each pair of molecules related by an inversion centre shows a π-interaction of 3.4 (1) Å between their planar furo[3,2-*c*]coumarin fragments. The substituent phenyl rings, despite their out-of-plane rotation, are directed far from the flat central molecular bulk and do not obstacle the strong π-stacking in the formation of the centrosymmetric couple, remaing at its surface as the co-crystallized methanol. Therefore crystal packing is constituted by dimeric units interconnected by several weak hydrogen bonds involving the O atoms.

Here, as well as already found in the phenyl-furo[3,2-*c*]chromen-4-one (Bruno *et al.*, 2001) and in other related coumarin compounds reported in the October 2004 (Version 5.27) release of CSD (Allen, 2002), an important asymmetry in the O—C—O bond angles has been detected [β = O(1)—C(2)—O(11), 117.4 (2)° and α = C(3)—C(2)—O(11), 125.1 (2)°] Δ(α-β) = 7.7 (2)°. Even more similar significant differences have been observed in other molecules containing the furan-5-one fragment, as we have already reported (Grassi *et al.*, 2002).

*Ab initio* and DFT calculations on some models have been performed in order to clarify the relevant observed asymmetry in the O—C—O bond angles of the cumarinic fragment. From these results we can conclude that the observed and calculated carbonyl distortion arises exclusively from electronic rather than steric factors. However further statistical and theoretical study are necessary to better clarify the real nature of this asymmetry.

### Experimental

Fig. 2.

We recently described a novel kind of *cyclo*-condensation process that converts two equivalents of a münchnone and a 1,3-dicarbonyl compound into a functionalized enol lactone containing a protected α-amino acid (Grassi *et al.*, 2003). Here we report structural characterization of the enol lactone 3 synthesized by 4,5-dimethyl-2-phenyl-1,3-oxazolium-5-late (DMPO) 2 and 4-hydroxy-6-methylcoumarin 1 (an unsymmetrical enolisable dicarbonyl substrate). Single crystals of 3 suitable for X-ray analysis were obtained by slow evaporation from a methanole solution.

In trying to get some insight regarding this important asymmetry in the O—C—O bond angle of the cumarinic fragment an *ab initio* and DFT calculations on some model compounds, such as the bicyclic 3,4-dyhidro-pyrano[4,3-*b*]pyran-2,5-dione, a series of 3-substituted pyran-2-one, the cumarine and 3-methyl-cumarine, were performed by using the basis set 6-31+G(d,p) in *GAUSSIAN98* (Frisch *et al.*, 1998). At all level of calculations this controversial asymmetry (Kokila *et al.*, 1996) around carbonilic C atom is consistent with the X-ray structural observations.

### Refinement

A suitable colourless single-crystal was mounted on a capillary glass fiber. The intensity data were collected at room temperature up to 2θ = 50° by using the ω-2θ scan technique with variable scan speed on a Siemens P4 4-cirlce diffractometer with graphite monochromated Mo *K*α radiation. Lattice parameters were obtained from least-squares refinement of the setting angles of 59 reflections with 14 < 2θ < 35°. Intensities were corrected for Lonrentz polarization and then for absorption effetcts. No crystal deterioration was revealed during irradiation. The structure, solved by standard Direct Methods, was completed and refined by a combination of least squares technique and Fourier Syntheses. Whereas several H atoms were located on final ΔF map, the H atoms were included in the refinement among the "riding model" method with the *X*—H bond geometry and the H isotropic displacement parameter depending on the parent atom *X*. The refinement, with all non H atoms anisotropic and carried out by the full matrix least-square technique, has included a parameter for extinction correction. The last difference Fourier map showed no significant density residuals. One terminal methyl (C16) appeared to be affected by a significant rotational disorder and its H's group has to be split over two symmetric positions with equal occupancy 0.5; the disorder of the methanol molecule causes its large thermal ellipsoids.

### Crystal data

**Table:**

C_28_H_23_NO_5_·CH_4_O	*F*(000) = 1024
*M**_r_* = 485.52	*D*_x_ = 1.305 Mg m^−^^3^
Monoclinic, *P*2_1_/*n*	Mo *K*α radiation, λ = 0.71073 Å
Hall symbol: -P 2yn	Cell parameters from 59 reflections
*a* = 10.7435 (14) Å	θ = 7.7–17.5°
*b* = 9.9056 (17) Å	µ = 0.09 mm^−^^1^
*c* = 23.298 (3) Å	*T* = 298 K
β = 94.817 (7)°	Irregular, colourless
*V* = 2470.7 (6) Å^3^	0.58 × 0.38 × 0.36 mm
*Z* = 4	

### Data collection

**Table:**

Siemens P4 diffractometer	2843 reflections with *I* > 2σ(*I*)
Radiation source: sealed tube	*R*_int_ = 0.036
graphite	θ_max_ = 25.0°, θ_min_ = 2.0°
ω scans	*h* = −12→12
Absorption correction: ψ scan (Kopfmann & Huber, 1968)	*k* = −1→11
*T*_min_ = 0.870, *T*_max_ = 0.968	*l* = −27→27
9535 measured reflections	3 standard reflections every 197 reflections
4347 independent reflections	intensity decay: none

### Refinement

**Table:**

Refinement on *F*^2^	Secondary atom site location: difference Fourier map
Least-squares matrix: full	Hydrogen site location: inferred from neighbouring sites
*R*[*F*^2^ > 2σ(*F*^2^)] = 0.049	H-atom parameters constrained
*wR*(*F*^2^) = 0.132	*w* = 1/[σ^2^(*F*_o_^2^) + (0.0533*P*)^2^ + 0.7913*P*] where *P* = (*F*_o_^2^ + 2*F*_c_^2^)/3
*S* = 1.01	(Δ/σ)_max_ = 0.001
4347 reflections	Δρ_max_ = 0.18 e Å^−^^3^
331 parameters	Δρ_min_ = −0.31 e Å^−^^3^
0 restraints	Extinction correction: *SHELXL97* (Sheldrick, 2008), Fc^*^=kFc[1+0.001xFc^2^λ^3^/sin(2θ)]^-1/4^
Primary atom site location: structure-invariant direct methods	Extinction coefficient: 0.0036 (8)

### Special details

**Table:**

Geometry. All e.s.d.'s (except the e.s.d. in the dihedral angle between two l.s. planes) are estimated using the full covariance matrix. The cell e.s.d.'s are taken into account individually in the estimation of e.s.d.'s in distances, angles and torsion angles; correlations between e.s.d.'s in cell parameters are only used when they are defined by crystal symmetry. An approximate (isotropic) treatment of cell e.s.d.'s is used for estimating e.s.d.'s involving l.s. planes.
Refinement. Refinement of *F*^2^ against ALL reflections. The weighted *R*-factor *wR* and goodness of fit *S* are based on *F*^2^, conventional *R*-factors *R* are based on *F*, with *F* set to zero for negative *F*^2^. The threshold expression of *F*^2^ > σ(*F*^2^) is used only for calculating *R*-factors(gt) *etc*. and is not relevant to the choice of reflections for refinement. *R*-factors based on *F*^2^ are statistically about twice as large as those based on *F*, and *R*-factors based on ALL data will be even larger.

### Fractional atomic coordinates and isotropic or equivalent isotropic displacement parameters (Å^2^)

**Table:**

	*x*	*y*	*z*	*U*_iso_*/*U*_eq_	Occ. (<1)
O1	0.50718 (15)	0.77955 (19)	0.49310 (6)	0.0560 (5)	
C2	0.6021 (2)	0.8610 (3)	0.51636 (10)	0.0490 (6)	
C3	0.64762 (19)	0.8378 (2)	0.57594 (9)	0.0440 (6)	
C4	0.5962 (2)	0.7392 (2)	0.60580 (9)	0.0456 (6)	
C5	0.4958 (2)	0.6556 (3)	0.58143 (10)	0.0483 (6)	
C6	0.4409 (2)	0.5503 (3)	0.61028 (12)	0.0588 (7)	
H6	0.4684	0.5327	0.6485	0.071*	
C7	0.3471 (2)	0.4720 (3)	0.58354 (14)	0.0675 (8)	
C8	0.3087 (2)	0.5014 (3)	0.52619 (15)	0.0735 (9)	
H8	0.2451	0.4500	0.5075	0.088*	
C9	0.3612 (2)	0.6034 (3)	0.49630 (12)	0.0655 (8)	
H9	0.3340	0.6203	0.4580	0.079*	
C10	0.4550 (2)	0.6802 (3)	0.52418 (10)	0.0512 (6)	
O11	0.64195 (16)	0.9454 (2)	0.48534 (7)	0.0649 (5)	
C12	0.75139 (19)	0.9241 (3)	0.60261 (9)	0.0442 (6)	
H12	0.7380	1.0154	0.5872	0.053*	
C13	0.7374 (2)	0.9318 (3)	0.66888 (9)	0.0447 (6)	
C14	0.7351 (2)	0.7859 (3)	0.68848 (9)	0.0491 (6)	
O15	0.64021 (15)	0.70590 (17)	0.66098 (6)	0.0538 (4)	
C16	0.2891 (3)	0.3579 (3)	0.61477 (16)	0.0964 (11)	
H16A	0.2461	0.2984	0.5873	0.145*	0.50
H16B	0.2310	0.3938	0.6399	0.145*	0.50
H16C	0.3533	0.3087	0.6370	0.145*	0.50
H16D	0.3075	0.3689	0.6555	0.145*	0.50
H16E	0.3226	0.2735	0.6029	0.145*	0.50
H16F	0.2003	0.3585	0.6058	0.145*	0.50
C17	0.8802 (2)	0.8790 (3)	0.58752 (9)	0.0493 (6)	
C18	0.9676 (2)	0.9762 (3)	0.57662 (11)	0.0684 (8)	
H18	0.9468	1.0670	0.5789	0.082*	
C19	1.0865 (3)	0.9398 (4)	0.56224 (12)	0.0835 (10)	
H19	1.1443	1.0064	0.5552	0.100*	
C20	1.1177 (3)	0.8095 (5)	0.55848 (13)	0.0848 (11)	
H20	1.1971	0.7857	0.5490	0.102*	
C21	1.0328 (3)	0.7111 (4)	0.56862 (13)	0.0832 (10)	
H21	1.0546	0.6207	0.5658	0.100*	
C22	0.9136 (2)	0.7461 (3)	0.58313 (11)	0.0633 (7)	
H22	0.8563	0.6787	0.5899	0.076*	
C23	0.8437 (2)	1.0061 (3)	0.70278 (10)	0.0583 (7)	
H23A	0.8475	1.0973	0.6891	0.087*	
H23B	0.9212	0.9613	0.6977	0.087*	
H23C	0.8294	1.0067	0.7429	0.087*	
N24	0.61573 (15)	0.9977 (2)	0.67699 (7)	0.0444 (5)	
C25	0.5853 (2)	1.1225 (3)	0.64515 (11)	0.0613 (7)	
H25A	0.6605	1.1728	0.6412	0.092*	
H25B	0.5290	1.1756	0.6657	0.092*	
H25C	0.5467	1.1007	0.6076	0.092*	
C26	0.5548 (2)	0.9634 (3)	0.72315 (10)	0.0490 (6)	
O27	0.59794 (16)	0.8770 (2)	0.75754 (7)	0.0659 (5)	
C28	0.4305 (2)	1.0259 (3)	0.73030 (10)	0.0502 (6)	
C29	0.3308 (2)	1.0086 (3)	0.68954 (12)	0.0716 (8)	
H29	0.3414	0.9615	0.6558	0.086*	
C30	0.2151 (2)	1.0614 (4)	0.69901 (14)	0.0843 (10)	
H30	0.1477	1.0472	0.6719	0.101*	
C31	0.1984 (3)	1.1333 (4)	0.74701 (14)	0.0807 (9)	
H31	0.1206	1.1700	0.7526	0.097*	
C32	0.2965 (3)	1.1516 (4)	0.78720 (13)	0.0806 (9)	
H32	0.2856	1.2013	0.8203	0.097*	
C33	0.4116 (2)	1.0973 (3)	0.77930 (11)	0.0657 (8)	
H33	0.4773	1.1091	0.8075	0.079*	
O34	0.81109 (15)	0.7302 (2)	0.72014 (7)	0.0637 (5)	
C34	0.5741 (5)	0.8527 (5)	0.8947 (2)	0.1397 (17)	
H34A	0.5325	0.7866	0.8698	0.210*	
H34B	0.5214	0.9305	0.8969	0.210*	
H34C	0.5915	0.8150	0.9325	0.210*	
O35	0.6784 (3)	0.8881 (7)	0.87417 (13)	0.208 (2)	
H35	0.6675	0.8947	0.8390	0.312*	

### Atomic displacement parameters (Å^2^)

**Table:**

	*U*^11^	*U*^22^	*U*^33^	*U*^12^	*U*^13^	*U*^23^
O1	0.0512 (9)	0.0702 (12)	0.0453 (9)	0.0038 (9)	−0.0040 (7)	−0.0002 (9)
C2	0.0414 (12)	0.0628 (17)	0.0433 (12)	0.0088 (12)	0.0060 (10)	−0.0008 (13)
C3	0.0388 (11)	0.0561 (15)	0.0378 (11)	0.0061 (11)	0.0068 (9)	0.0007 (11)
C4	0.0450 (12)	0.0535 (15)	0.0383 (11)	0.0068 (12)	0.0044 (9)	−0.0019 (11)
C5	0.0400 (12)	0.0528 (15)	0.0533 (14)	0.0048 (11)	0.0110 (10)	−0.0051 (12)
C6	0.0509 (14)	0.0589 (17)	0.0680 (16)	0.0005 (13)	0.0135 (12)	−0.0055 (14)
C7	0.0489 (15)	0.0576 (18)	0.099 (2)	−0.0006 (14)	0.0214 (15)	−0.0127 (17)
C8	0.0433 (14)	0.074 (2)	0.102 (2)	−0.0026 (15)	0.0015 (15)	−0.0291 (19)
C9	0.0494 (15)	0.076 (2)	0.0696 (17)	0.0070 (15)	−0.0045 (13)	−0.0197 (16)
C10	0.0397 (12)	0.0585 (17)	0.0553 (14)	0.0072 (12)	0.0041 (11)	−0.0069 (13)
O11	0.0640 (11)	0.0864 (14)	0.0446 (9)	−0.0029 (10)	0.0072 (8)	0.0148 (10)
C12	0.0405 (12)	0.0535 (15)	0.0390 (11)	0.0011 (11)	0.0058 (9)	0.0027 (11)
C13	0.0387 (12)	0.0575 (16)	0.0382 (11)	0.0040 (11)	0.0043 (9)	−0.0002 (11)
C14	0.0447 (12)	0.0655 (17)	0.0373 (11)	0.0055 (13)	0.0060 (10)	0.0006 (12)
O15	0.0619 (10)	0.0573 (11)	0.0417 (8)	−0.0035 (9)	0.0011 (7)	0.0069 (8)
C16	0.076 (2)	0.076 (2)	0.141 (3)	−0.0171 (18)	0.029 (2)	−0.003 (2)
C17	0.0421 (12)	0.0686 (18)	0.0378 (12)	−0.0006 (13)	0.0079 (10)	−0.0013 (12)
C18	0.0584 (16)	0.086 (2)	0.0632 (16)	−0.0079 (16)	0.0182 (13)	−0.0048 (16)
C19	0.0550 (17)	0.129 (3)	0.0698 (19)	−0.015 (2)	0.0224 (14)	−0.009 (2)
C20	0.0500 (16)	0.138 (3)	0.0687 (19)	0.014 (2)	0.0170 (14)	−0.014 (2)
C21	0.0671 (19)	0.103 (3)	0.081 (2)	0.027 (2)	0.0170 (16)	−0.0044 (19)
C22	0.0546 (15)	0.073 (2)	0.0631 (16)	0.0080 (15)	0.0110 (12)	−0.0009 (14)
C23	0.0463 (13)	0.0765 (19)	0.0518 (13)	−0.0025 (14)	0.0015 (11)	−0.0070 (14)
N24	0.0397 (10)	0.0542 (12)	0.0397 (9)	0.0036 (9)	0.0068 (8)	0.0038 (9)
C25	0.0583 (15)	0.0686 (19)	0.0586 (15)	0.0116 (14)	0.0158 (12)	0.0123 (14)
C26	0.0455 (13)	0.0591 (16)	0.0427 (12)	0.0009 (12)	0.0049 (10)	−0.0023 (12)
O27	0.0669 (11)	0.0826 (14)	0.0501 (10)	0.0178 (11)	0.0156 (8)	0.0156 (10)
C28	0.0417 (12)	0.0607 (16)	0.0490 (13)	−0.0030 (12)	0.0091 (10)	0.0024 (12)
C29	0.0502 (15)	0.100 (2)	0.0642 (16)	−0.0008 (16)	0.0047 (12)	−0.0214 (17)
C30	0.0403 (15)	0.128 (3)	0.084 (2)	−0.0023 (17)	−0.0005 (14)	−0.011 (2)
C31	0.0505 (16)	0.113 (3)	0.081 (2)	0.0139 (17)	0.0204 (15)	−0.002 (2)
C32	0.0637 (18)	0.107 (3)	0.0732 (19)	0.0124 (18)	0.0176 (15)	−0.0207 (18)
C33	0.0503 (14)	0.091 (2)	0.0564 (15)	0.0016 (15)	0.0070 (12)	−0.0154 (15)
O34	0.0554 (10)	0.0799 (13)	0.0549 (10)	0.0163 (10)	−0.0014 (8)	0.0164 (10)
C34	0.159 (4)	0.135 (4)	0.128 (4)	0.009 (4)	0.024 (3)	0.029 (3)
O35	0.140 (3)	0.398 (7)	0.0822 (19)	0.048 (4)	−0.0186 (19)	0.013 (3)

### Geometric parameters (Å, °)

**Table:**

O1—C10	1.369 (3)	C18—H18	0.9300
O1—C2	1.376 (3)	C19—C20	1.339 (5)
C2—O11	1.206 (3)	C19—H19	0.9300
C2—C3	1.451 (3)	C20—C21	1.369 (5)
C3—C4	1.344 (3)	C20—H20	0.9300
C3—C12	1.498 (3)	C21—C22	1.395 (4)
C4—O15	1.372 (3)	C21—H21	0.9300
C4—C5	1.438 (3)	C22—H22	0.9300
C5—C10	1.390 (3)	C23—H23A	0.9600
C5—C6	1.398 (3)	C23—H23B	0.9600
C6—C7	1.379 (4)	C23—H23C	0.9600
C6—H6	0.9300	N24—C26	1.349 (3)
C7—C8	1.396 (4)	N24—C25	1.464 (3)
C7—C16	1.507 (4)	C25—H25A	0.9600
C8—C9	1.375 (4)	C25—H25B	0.9600
C8—H8	0.9300	C25—H25C	0.9600
C9—C10	1.381 (4)	C26—O27	1.236 (3)
C9—H9	0.9300	C26—C28	1.493 (3)
C12—C17	1.524 (3)	C28—C33	1.373 (3)
C12—C13	1.566 (3)	C28—C29	1.382 (3)
C12—H12	0.9800	C29—C30	1.383 (4)
C13—N24	1.488 (3)	C29—H29	0.9300
C13—C14	1.516 (4)	C30—C31	1.351 (4)
C13—C23	1.523 (3)	C30—H30	0.9300
C14—O34	1.189 (3)	C31—C32	1.362 (4)
C14—O15	1.403 (3)	C31—H31	0.9300
C16—H16A	0.9600	C32—C33	1.374 (4)
C16—H16B	0.9600	C32—H32	0.9300
C16—H16C	0.9600	C33—H33	0.9300
C16—H16D	0.9600	C34—O35	1.302 (5)
C16—H16E	0.9600	C34—H34A	0.9600
C16—H16F	0.9600	C34—H34B	0.9600
C17—C22	1.371 (4)	C34—H34C	0.9600
C17—C18	1.382 (4)	O35—H35	0.8200
C18—C19	1.395 (4)		
			
C10—O1—C2	122.43 (19)	H16D—C16—H16F	109.5
O11—C2—O1	117.3 (2)	H16E—C16—H16F	109.5
O11—C2—C3	125.1 (2)	C22—C17—C18	118.0 (2)
O1—C2—C3	117.6 (2)	C22—C17—C12	123.1 (2)
C4—C3—C2	119.4 (2)	C18—C17—C12	118.8 (2)
C4—C3—C12	121.6 (2)	C17—C18—C19	120.9 (3)
C2—C3—C12	119.0 (2)	C17—C18—H18	119.5
C3—C4—O15	122.4 (2)	C19—C18—H18	119.5
C3—C4—C5	122.8 (2)	C20—C19—C18	120.3 (3)
O15—C4—C5	114.8 (2)	C20—C19—H19	119.9
C10—C5—C6	118.7 (2)	C18—C19—H19	119.9
C10—C5—C4	116.3 (2)	C19—C20—C21	120.1 (3)
C6—C5—C4	124.9 (2)	C19—C20—H20	120.0
C7—C6—C5	121.6 (3)	C21—C20—H20	120.0
C7—C6—H6	119.2	C20—C21—C22	120.3 (3)
C5—C6—H6	119.2	C20—C21—H21	119.9
C6—C7—C8	117.5 (3)	C22—C21—H21	119.9
C6—C7—C16	121.4 (3)	C17—C22—C21	120.5 (3)
C8—C7—C16	121.2 (3)	C17—C22—H22	119.8
C9—C8—C7	122.5 (3)	C21—C22—H22	119.8
C9—C8—H8	118.8	C13—C23—H23A	109.5
C7—C8—H8	118.8	C13—C23—H23B	109.5
C8—C9—C10	118.8 (3)	H23A—C23—H23B	109.5
C8—C9—H9	120.6	C13—C23—H23C	109.5
C10—C9—H9	120.6	H23A—C23—H23C	109.5
O1—C10—C9	117.6 (2)	H23B—C23—H23C	109.5
O1—C10—C5	121.5 (2)	C26—N24—C25	121.0 (2)
C9—C10—C5	120.9 (3)	C26—N24—C13	118.76 (19)
C3—C12—C17	113.34 (19)	C25—N24—C13	117.86 (18)
C3—C12—C13	107.90 (17)	N24—C25—H25A	109.5
C17—C12—C13	113.96 (17)	N24—C25—H25B	109.5
C3—C12—H12	107.1	H25A—C25—H25B	109.5
C17—C12—H12	107.1	N24—C25—H25C	109.5
C13—C12—H12	107.1	H25A—C25—H25C	109.5
N24—C13—C14	110.10 (18)	H25B—C25—H25C	109.5
N24—C13—C23	110.53 (19)	O27—C26—N24	120.7 (2)
C14—C13—C23	109.6 (2)	O27—C26—C28	120.3 (2)
N24—C13—C12	107.74 (16)	N24—C26—C28	118.9 (2)
C14—C13—C12	104.82 (19)	C33—C28—C29	118.3 (2)
C23—C13—C12	113.90 (18)	C33—C28—C26	120.2 (2)
O34—C14—O15	117.1 (2)	C29—C28—C26	121.4 (2)
O34—C14—C13	127.0 (2)	C28—C29—C30	119.9 (3)
O15—C14—C13	115.49 (19)	C28—C29—H29	120.0
C4—O15—C14	118.16 (18)	C30—C29—H29	120.0
C7—C16—H16A	109.5	C31—C30—C29	121.0 (3)
C7—C16—H16B	109.5	C31—C30—H30	119.5
H16A—C16—H16B	109.5	C29—C30—H30	119.5
C7—C16—H16C	109.5	C30—C31—C32	119.4 (3)
H16A—C16—H16C	109.5	C30—C31—H31	120.3
H16B—C16—H16C	109.5	C32—C31—H31	120.3
C7—C16—H16D	109.5	C31—C32—C33	120.6 (3)
H16A—C16—H16D	141.1	C31—C32—H32	119.7
H16B—C16—H16D	56.3	C33—C32—H32	119.7
H16C—C16—H16D	56.3	C28—C33—C32	120.7 (3)
C7—C16—H16E	109.5	C28—C33—H33	119.6
H16A—C16—H16E	56.3	C32—C33—H33	119.6
H16B—C16—H16E	141.1	O35—C34—H34A	109.5
H16C—C16—H16E	56.3	O35—C34—H34B	109.5
H16D—C16—H16E	109.5	H34A—C34—H34B	109.5
C7—C16—H16F	109.5	O35—C34—H34C	109.5
H16A—C16—H16F	56.3	H34A—C34—H34C	109.5
H16B—C16—H16F	56.3	H34B—C34—H34C	109.5
H16C—C16—H16F	141.1	C34—O35—H35	109.5
			
C10—O1—C2—O11	179.8 (2)	N24—C13—C14—O15	57.6 (2)
C10—O1—C2—C3	0.6 (3)	C23—C13—C14—O15	179.33 (17)
O11—C2—C3—C4	−179.2 (2)	C12—C13—C14—O15	−58.1 (2)
O1—C2—C3—C4	−0.1 (3)	C3—C4—O15—C14	5.6 (3)
O11—C2—C3—C12	0.5 (3)	C5—C4—O15—C14	−177.71 (18)
O1—C2—C3—C12	179.67 (19)	O34—C14—O15—C4	−144.9 (2)
C2—C3—C4—O15	175.6 (2)	C13—C14—O15—C4	28.2 (3)
C12—C3—C4—O15	−4.2 (3)	C3—C12—C17—C22	−38.0 (3)
C2—C3—C4—C5	−0.8 (3)	C13—C12—C17—C22	85.9 (3)
C12—C3—C4—C5	179.5 (2)	C3—C12—C17—C18	140.8 (2)
C3—C4—C5—C10	1.1 (3)	C13—C12—C17—C18	−95.3 (3)
O15—C4—C5—C10	−175.52 (19)	C22—C17—C18—C19	−0.7 (4)
C3—C4—C5—C6	178.7 (2)	C12—C17—C18—C19	−179.5 (2)
O15—C4—C5—C6	2.0 (3)	C17—C18—C19—C20	0.3 (5)
C10—C5—C6—C7	−0.7 (4)	C18—C19—C20—C21	0.1 (5)
C4—C5—C6—C7	−178.2 (2)	C19—C20—C21—C22	−0.2 (5)
C5—C6—C7—C8	0.1 (4)	C18—C17—C22—C21	0.6 (4)
C5—C6—C7—C16	179.5 (2)	C12—C17—C22—C21	179.4 (2)
C6—C7—C8—C9	0.4 (4)	C20—C21—C22—C17	−0.1 (4)
C16—C7—C8—C9	−178.9 (3)	C14—C13—N24—C26	35.9 (3)
C7—C8—C9—C10	−0.4 (4)	C23—C13—N24—C26	−85.3 (3)
C2—O1—C10—C9	−179.3 (2)	C12—C13—N24—C26	149.6 (2)
C2—O1—C10—C5	−0.3 (3)	C14—C13—N24—C25	−161.6 (2)
C8—C9—C10—O1	178.8 (2)	C23—C13—N24—C25	77.2 (2)
C8—C9—C10—C5	−0.1 (4)	C12—C13—N24—C25	−47.8 (3)
C6—C5—C10—O1	−178.3 (2)	C25—N24—C26—O27	−163.2 (2)
C4—C5—C10—O1	−0.6 (3)	C13—N24—C26—O27	−1.3 (3)
C6—C5—C10—C9	0.7 (3)	C25—N24—C26—C28	20.1 (3)
C4—C5—C10—C9	178.4 (2)	C13—N24—C26—C28	−177.9 (2)
C4—C3—C12—C17	98.8 (2)	O27—C26—C28—C33	61.0 (4)
C2—C3—C12—C17	−80.9 (3)	N24—C26—C28—C33	−122.4 (3)
C4—C3—C12—C13	−28.3 (3)	O27—C26—C28—C29	−116.1 (3)
C2—C3—C12—C13	151.9 (2)	N24—C26—C28—C29	60.5 (3)
C3—C12—C13—N24	−62.3 (2)	C33—C28—C29—C30	−0.8 (4)
C17—C12—C13—N24	170.9 (2)	C26—C28—C29—C30	176.3 (3)
C3—C12—C13—C14	55.0 (2)	C28—C29—C30—C31	1.9 (5)
C17—C12—C13—C14	−71.8 (2)	C29—C30—C31—C32	−1.4 (5)
C3—C12—C13—C23	174.7 (2)	C30—C31—C32—C33	−0.2 (5)
C17—C12—C13—C23	47.9 (3)	C29—C28—C33—C32	−0.8 (4)
N24—C13—C14—O34	−130.1 (2)	C26—C28—C33—C32	−177.9 (3)
C23—C13—C14—O34	−8.3 (3)	C31—C32—C33—C28	1.3 (5)
C12—C13—C14—O34	114.3 (2)		

### Hydrogen-bond geometry (Å, °)

**Table:**

*D*—H···*A*	*D*—H	H···*A*	*D*···*A*	*D*—H···*A*
O35—H35···O27	0.82	1.99	2.784 (3)	163
C9—H9···O35^i^	0.93	2.46	3.320 (4)	153
C19—H19···O11^ii^	0.93	2.60	3.403 (3)	144

Symmetry codes: (i) *x*−1/2, −*y*+3/2, *z*−1/2; (ii) −*x*+2, −*y*+2, −*z*+1.
